# Leukemogenesis and ageing: ‘fit for transformation’ ?

**DOI:** 10.18632/aging.100274

**Published:** 2011-02-22

**Authors:** Mel Greaves

**Affiliations:** Section of Haemato-Oncology, The Institute of Cancer Research, Sutton, UK

There is longstanding recognition of the striking association between ageing in humans and the incidence rates of many common types of cancer. The biological basis of this link is likely to be multi-factorial including both age-associated decline in anti-oncogenic functions, such as repair of oxidative damage to DNA, timing of etiologic exposures and/or protracted time required for accumulation of a full set of oncogenic mutations [1,2].

Acute leukemias have an unusual age-associated incidence with a peak at a young age (2-5 years) followed by a later increasing incidence with age in adults. In this instance, however, the biological and genetic subtypes of leukemia are distinct at different ages [3].

*BCR-ABL1* gene fusion is the result of the ‘Philadelphia’ chromosome - the first specific genetic abnormality described in cancer and which is a translocation between chromosomes 9 and 22 [4]. Two versions of the fusion exist involving different exons of *BCR* and generating two different sized proteins: *p210 BCR-ABL1* predominantly associated with chronic myeloid leukemia and *p190 BCR-ABL1* linked to B cell precursor acute lymphoblastic leukemia (ALL). Both sub-types of leukemia have increased incidence with age.

So what might be the biological basis of the age-associated incidence of B-ALL with *p190 BCR-ABL1*? Vicente-Dueñas et al [5] designed a novel experiment to address this question which conveniently bypassed uncertainties over required exposures for the natural disease and age-associated alterations in the host tissue micro-environment. Using an inducible p190 BCR-ABL transgene, they found that ‘old’ (= 20 months) cells develop leukemias at a much faster rate (~2x) than young' (= 4 months) cells when transplanted into recipients of the same age (4 weeks). The key to the experiment is that *BCR-ABL1* expression is not activated until after the transplant.

Although all mice eventually developed ALL, this result is compatible with the notion that ‘old’ B progenitors have some competitive advantage when expressing *BCR-ABL1*. What might this be? A plausible explanation is offered in another paper recently published by Henry et al [6]. This group have previously suggested that age-related fitness decline in stem and progenitor cells might increase selective pressure for oncogenic mutations [7]. In their new study [6], they also evaluated the impact of age on the transformability of hematopoietic cells by *p190 BCR-ABL1*. In their case, this was performed by transfecting ‘young’ (2 months) versus ‘old’ (22-24 months) bone marrow cells with a *p190 BCR-ABL* retrovirus and transplanting these into recipients which were selected to provide either ‘young’ (2 months) or ‘old’ (22-24 months) competitor normal cells. They observed higher rates of leukemia (up to 200 days post-transplant) with transfer of ‘older’ cells. More rapid onset of leukemia in aged *p190 BCR-ABL1* cells or more efficient leukemogenesis probably reflects more ‘target’ cells being transformed which, in turn, would increase the probability of the acquisition of secondary genetic abnormalities such as *IKZF1* deletion which, at least for leukemia in patients with *BCR-ABL1*+ ALL, appear to be critical for malignant progression [8]. This interpretation accords with the observation of Henry et al [6] that ‘older’ mouse cells transfected with *BCR-ABL1* undergo more rapid polyclonal expansion early after transplantation.

Additionally, Henry et al [6] observed that ‘young’ competitor cells in the recipient had a restraining impact on the leukemogenic efficiency of ‘old’ transformed cells, indicating that the age-associated leukemogenesis involves both cell intrinsic (or autonomous) and extrinsic factors perhaps related to competitive occupancy of critical bone marrow niches. Henry et al argue that *BCR-ABL1* actually has more potent selective advantage in older cells because it restores defective kinase signalling in ageing cells. They provide supportive evidence for this by showing increased expansion of young *BCR-ABL1*+ B cell progenitors under conditions of reduced kinase signalling via the regulatory molecule IL7 [6].

These data are compelling but one alternative explanation might also be considered. B cell lymphopoiesis declines dramatically with age in both mice and humans and this inevitably produces substantial changes in the cellular architecture of bone marrow [9]. Transforming functions of oncogenes, including leukemic fusion genes, in very cell context dependent [10]. It is possible that it is not only some qualitative feature of the target cell that changes with age but the number of the appropriate cells available for transformation.

With ionizing radiation-induced ALL, children [11] or young mice [12] are more vulnerable, as are young post-adolescent females with respect to irradiation-induced breast cancer [13]. This is most easily interpreted as reflecting age-associated changes in the number and proliferative activity of the particular stem and progenitor cells that provide the vulnerable ‘targets’ for transformation.

Whatever the mechanisms, or combination of mechanisms, involved, these two new papers add a new dimension to our understanding of the complexity of age-associated leukemogenesis and, perhaps, cancer in general.
